# Criminal Carelessness

**Published:** 1869-08-15

**Authors:** 


### EDITORIAL.

### *Criminal Carelessness.*

The morning papers of August 2d contain the following verdict of a coroner's jury:

"We, the jury, find that the said George H. Deming came to his death from an overdose of *aconite*, administered for brandy by one J. J. Harrington, druggist, on the corner of Illinois and Rush streets; and from the testimony adduced, we, the jury, believe that the said Harrington was guilty of criminal carelessness in not having his bottles plainly marked, and in their proper places, through which means the mistake occurred which led to this fatal result."

Another sample of "crowner's quest law," to which an admirable parallel may be found in Hamlet, act V, scene I. The man is dead, "the coroner hath sat on him, and finds it *misplaced bottles.* Nothing whatever is said of the criminality of tampering with human life by persons who can not, upon any hypothesis, be supposed to know anything about the principles of medicine. Had the brandy been administered in accordance with the intentions of the druggist, the verdict of the jury, *criminal carelessness*, would have been quite as appropriate.

We have no desire to add one jot to the punishment which this man's conscience will inflict upon him, and believe that it alone will be a sufficient atonement for his fault. Not upon his shoulders alone should the lash be laid, but upon those of the public which sanction and sustain the practice of tampering with human health and life, prevalent amongst some (we are glad to say, not the best) apothecaries in this city. What would have been thought of the apothecary who should have attempted to amputate the man's leg, had it been accidentally injured, and thereby occsioned his death? Would the verdict in such a case have been "*criminal carelessness*" in not properly ligating the vessels? or would it have been willfully endangering human life in presuming to attempt what he knew he could not perform? If this had been the first case of the sort, there would be some apology for the evasive chnracter of the verdict above quoted; but when it is well known that drugs of the most deadly nature are daily and hourly prescribed and sold by apothecaries' clerks in this city, upon no other warrant than their own natural judgment; when it is also known that a large proportion of the suicides are thus supplied with the means of self-murder, it seems much more appropriate that their own verdict, "criminal carelessness," should be found against the coroner's jury by that higher tribunal, public opinion.

